# Treatment Strategies for Metastatic Soft Tissue Sarcomas

**DOI:** 10.3390/cancers13071722

**Published:** 2021-04-06

**Authors:** Lisette M. Wiltink, Rick L. M. Haas, Hans Gelderblom, Michiel A. J. van de Sande

**Affiliations:** 1 Department of Radiation Oncology, Leiden University Medical Center, 2300 RC Leiden, The Netherlands; r.haas@nki.nl; 2 Department of Radiation Oncology, Netherlands Cancer Institute, 1006 BE Amsterdam, The Netherlands; 3 Department of Medical Oncology, Leiden University Medical Center, 2300 RC Leiden, The Netherlands; A.J.Gelderblom@lumc.nl; 4 Department of Orthopedic Surgery, Leiden University Medical Center, 2300 RC Leiden, The Netherlands; M.A.J.van_de_Sande@lumc.nl

Soft tissue sarcomas (STS) are a diverse group of rare tumors of mesenchymal origin with different clinical, histologic and molecular characteristics [1]. While most patients have localized disease at diagnosis, about 10% present with synchronous metastatic disease [2]. Although myxoid and dedifferentiated liposarcomas metastasize to limb, soft tissue, bone, retroperitoneum and the pelvis, the most common site for metastasis of other sarcomas is the lungs. About 50% of high-grade STS patients eventually develop pulmonary metastases. Within 70% of these patients, the lungs remain the only organ with metastatic disease [3]. Patients with metastasized STS generally have a poor prognosis, with a median survival of 12 months [4]. However, for example, in the PICASSO III study, an improved median overall survival of 16–17 months is reported after systemic treatment [5]. 

The current ESMO clinical practice guideline recommends mainly systemic treatment for advanced and metastatic STS [6]. Anthracyclines (e.g., doxorubicin) are advised as first-line treatment. Combined treatment with ifosfamide can lead to higher tumor response rates and a prolonged progression free survival [7]. Clearly the anticipated side effects of this systemic therapy should be balanced with the expected benefits, in an effort to decrease the anticipated side effects of adding ifosfamide to anthracyclines. Dacarbazine could be considered, instead of ifosfamide, for leiomyosarcomas and solitary fibrous tumors [8,9]. Doxorubicin, in combination with the anti-PDGFRA biological olaratumab, has been investigated, but failed to show a survival benefit as compared to doxorubicin alone [10]. For several metastatic STS subtypes, such as angiosarcomas, leiomyosarcomas and dermatofibrosarcomas protuberans, histotype tailored chemotherapeutic regimes are recommended [6]. For other STS subtypes, histotype tailored chemotherapy was less effective than standard chemotherapy [11]. Further-line therapies, such as molecular targeted agents for selected subtypes, can be considered for patients with a progressive disease [6,12]. 

Surgery is generally only advised for metachronous lung metastasis, with a disease-free interval for more than one year, if the lesions are located in the lungs alone, and if all lesions could be removed [6,13]. The benefit of (neo)adjuvant chemotherapy in a metastasectomy setting is not yet clear, as a randomized study on neoadjuvant chemotherapy before lung metastasectomy has been initiated (EORTC 62933), but was prematurely stopped [14]. In a retrospective cohort comparing metastasectomy or chemotherapy, followed by metastasectomy, no difference in disease specific or progression free survival was found; however, an improved progression free survival was reported in patients with a good (complete) histological response following chemotherapy [15]. Radiological response was found to be a prognostic factor for improved progression free survival and disease specific survival [15] 

In very selected cases, surgery, stereotactic radiotherapy, or ablation of metastasis could be considered without chemotherapy [6]. Recently, an improved survival, from 10.5 to 38.9 months, was found in soft tissue sarcoma patients after pulmonary metastasectomy alone. In multivariate analysis, metastasectomy was found to be the most important prognostic factor for survival [3]. In a cohort comparing patients with and without metastasectomy, a significantly improved 10 year overall survival of 17% versus 3% was found after surgery, leading to a median overall survival of 3.3 years compared to 0.9 years without surgery [16]. Furthermore, others studies have shown a better overall survival after metastasectomy [17,18]. Moreover, Wigge et al reported that repeated resections from different localizations was a strong predictor for improved survival [18]. Resection of oligometastatic hepatic metastasis can lead to an overall survival of 90.3% at one year, and, if a radical resection of the lesion could be achieved, extrahepatic disease should not be considered as a contraindication for resection [19]. Radiotherapy could also be used to locally control metastatic disease. Several cohorts show excellent outcomes, with minimal toxicity after stereotactic radiation therapy [20,21,22,23]. At a one year follow-up, a local control rate of 94% and an overall survival of 76% are reported [21]. Brachytherapy of metastasis with (125)I seeds is reported to improve progression free survival after a failure of first-line chemotherapy, from 3.6 to 7 months, compared to chemotherapy alone [24]. A few studies have been published about interventional radiology treatment options, such as radio frequent ablation. In one of these, ablation is shown to be effective for local metastatic control in leiomyosarcomas, with a local control rate at one year of 95.2% [25].

All studies focusing on local control of metastatic disease and survival are retrospective, which may lead to selection bias, as, typically, only selected patients are offered local therapy for metastases. Since STSs are relatively rare, it is difficult to design prospective patient cohorts, and even more difficult to include enough patient numbers in randomized controlled trials, in order to compare different treatment options in a homogeneous patient population. There is also reluctance from patients and physicians to randomize between local therapy and no local therapy, as there is almost always a center preference. For that reason, these trials are likely to fail due to poor recruitment. With the relatively poor prognosis of STS, quality of life during and after treatment is even regarded as more important; yet, in almost none of the studies is quality of life after treatment reported. 

Finally, another treatment option not yet discussed, is best supportive care, which always remains the alternative for patients unfit or unwilling to undergo local or systemic treatments. Short-course palliative radiotherapy could be part of this strategy by relieving pain caused by metastasis, with minimal toxicity [26].

In conclusion, palliative systemic treatment remains the standard treatment in metastatic disease, but, accumulating evidence shows improved overall survival after management of oligometastatic disease with surgery, radiotherapy, or interventional radiological ablation techniques. Traditionally, metastasectomy is the treatment of choice to achieve local control, but similar local control rates are reported after stereotactic radiotherapy techniques. It is likely that only a subset of STS patients will benefit from local metastatic treatment. Typically, these decisions can only be made in a multidisciplinary sarcoma tumor board in a reference center, as STS includes over 80 subtypes [6]. Specific treatment for each metastasized subtype will not be available in the near future. Well-designed studies should be encouraged to support subgroup analysis, as should quality of life outcome measures to help develop the best treatment strategy for each STS subtype.
